# An instant beverage rich in nutrients and secondary metabolites manufactured from stems and leaves of *Panax notoginseng*

**DOI:** 10.3389/fnut.2022.1058639

**Published:** 2022-12-07

**Authors:** Zhengwei Liang, Kunyi Liu, Ruoyu Li, Baiping Ma, Wei Zheng, Shengchao Yang, Guanghui Zhang, Yinhe Zhao, Junwen Chen, Ming Zhao

**Affiliations:** ^1^College of Agronomy and Biotechnology, Yunnan Agricultural University, Kunming, Yunnan, China; ^2^Yunnan Characteristic Plant Extraction Laboratory, Kunming, Yunnan, China; ^3^The Key Laboratory of Medicinal Plant Biology of Yunnan Province, National Local Joint Engineering Research Center on Germplasm Innovation and Utilization of Chinese Medicinal Materials in Southwestern, Kunming, Yunnan, China; ^4^College of Wuliangye Technology and Food Engineering, Yibin Vocational and Technical College, Yibin, Sichuan, China; ^5^College of Tea Science, Yunnan Agricultural University, Kunming, Yunnan, China; ^6^Beijing Institute of Radiation Medicine, Beijing, China

**Keywords:** antioxidant, nutrients, *Panax notoginseng*, polyphenol, saponin

## Abstract

**Introduction:**

Radix Notoginseng, one of the most famous Chinese traditional medicines, is the dried root of *Panax notoginseng* (Araliaceae). Stems and leaves of *P. notoginseng* (SLPN) are rich in secondary metabolites and nutrients, and authorized as a food resource, however, its utilization needs further research.

**Methods:**

A SLPN-instant beverage was manufactured from SLPN through optimization by response surface design with 21-fold of 48.50% ethanol for 39 h, and this extraction was repeated twice; the extraction solution was concentrated to 1/3 volume using a vacuum rotatory evaporator at 45°C, and then spray dried at 110°C. Nutritional components including 14 amino acids, ten mineral elements, 15 vitamins were detected in the SLPN-instant beverage; forty-three triterpenoid saponins, e.g., ginsenoside La, ginsenoside Rb3, notoginsenoside R1, and two flavonoid glycosides, as well as dencichine were identified by UPLC-MS.

**Results:**

The extraction rate of SLPN-instant beverage was 37.89 ± 0.02%. The majority nutrients were Gly (2.10 ± 0.63 mg/g), His (1.23 ± 0.07 mg/g), α-VE (18.89 ± 1.87 μg/g), β-VE (17.53 ± 1.98 μg/g), potassium (49.26 ± 2.70 mg/g), calcium (6.73 ± 0.27 mg/g). The total saponin of the SLPN-instant beverage was 403.05 ± 34.98 mg/g, majority was notoginsenoside Fd and with contents of 227 ± 2.02 mg/g. In addition, catechin and γ-aminobutyric acid were detected with levels of 24.57 ± 0.21 mg/g and 7.50 ± 1.85 mg/g, respectively. The SLPN-instant beverage showed good antioxidant activities with half maximal inhibitory concentration (IC_50_) for scavenging hydroxyl (OH^–^) radicals, superoxide anion (O^2–^) radicals, 1,1-diphenyl-2-picrylhydrazyl (DPPH) radicals and 2,2′-azino-bis(3-ethylbenzothiazoline-6-sulfonate) (ABTS+) radicals were 0.1954, 0.2314, 0.4083, and 0.3874 mg/mL, respectively.

**Conclusion:**

We optimized an analytical method for in depth analysis of the newly authorized food resource SLPN. Together, an instant beverage with antioxidant activity, rich in nutrients and secondary metabolites, was manufactured from SLPN, which may improve the utilization of SLPN.

## Introduction

*Panax notoginseng* (Burk) F.H. Chen is a perennial medicinal plant and a member of the Araliaceae family. The dried root of *P. notoginseng* is defined as Radix Notoginseng, which is a famous Chinese medicinal material and has been used for thousands of years in China (1). This herb is extensively used in more than 594 Chinese medicines (2) and the total saponins in Radix Notoginseng are thought to be pharmacological active components (3). The cultivation area of *P. notoginseng* has reached 1.03 × 10^4^ hm^2^ in China (4), and the total value of agricultural output has reached $ 0.87 billion (about ¥ 5.5 billion) in (5). Interestingly, total saponin including ginsenosides and notoginsenosides are also found in the stems and leaves of *P. notoginseng* (SLPN) (6), which is similar to that in roots of *P. notoginseng*. More than 20-million-kilogram SLPN are produced each year in China (7), but the utilization rate is <5%.

Stems and leaves of *P. notoginseng* contain secondary metabolites and nutrients, including polyphenols, flavonoids, volatile oil, dencichine, amino acids, vitamins, mineral elements etc. For example, SLPN were reported to contain vitamins (11.39 mg/100 g), crude fiber (8.33–20.45%), protein (9.63–15.18%), and fat (0.46–0.82%) (8). It mineral elements contents include zinc (Zn) (31.99–328.78 mg/kg), iron (Fe) (126.06–832.80 mg/kg), manganese (Mn) (102.58–431.18 mg/kg), calcium (Ca) (9.35–16.95 g/kg) and magnesium (Mg) (2.41–3.59 g/kg) (9). Besides, SLPN also contains abundant secondary metabolites and the total saponins in SLPN is 5–7% (10). To date, 945 ginsenosides have been identified in SLPN (11), such as notoginsenoside R_1_, ginsenoside Rb_1_, ginsenoside Rb_2_, notoginsenoside R_2_, notoginsenoside Fa, ginsenosides Rg_1_, ginsenosides Rg_3_, ginsenosides Rb_3_, ginsenosides Rc, ginsenosides Rd and the gypenoside family (12). The total sum content of notoginsenoside R_1_, ginsenosides Rb_1_, ginsenosides Rb_2_ amounts to 10.68 ± 0.97 mg/g in SLPN, and this is more than half of that in the roots (6). Two polyphenolic compounds, quercetin and gallic acid, are detected in SLPN with an average content of 0.16 and 0.02%, respectively (13). Therefore, SLPN are rich in nutritional components and secondary metabolites valued deeply for application.

Fortunately, the local food safety standard (DBS53/024 - 2017) for SLPN has been authenticated by the Health and Family Planning Commission of Yunnan province, China, and nowadays SLPN has been authorized as a food resource (14). It has been reported that vigorous ginsenoside transformation occurs in SLPN processed by sun-air drying and hot-air drying at 50^°^C, but not by shade-air drying, hot-air drying at 25^°^C and steaming prior to drying (15). Up to now, Radix Notoginseng saponins shows good antioxidant activity (3). Steamed Radix Notoginseng displayed a significant increase both in cell viability and oxygen radical absorption capacity values (16). Several foods containing ginsenosides which are extracted from SLPN have been on the market, e.g., ginsenoside Rb_3_ herbal teas (17), *P*. *notoginseng* stems and leaves extract candy (18), stem-leaves of *P*. *notoginseng* ginsenosides healthful-foods (6). Additionally, there is a Chinese medicine entitled by *Qiyeshen’an* tablets containing total saponin extracted from SLPN (19). The extraction methods of SLPN nutrition and secondary metabolites include hot water or high concentration hot ethanol extraction, ultrasonic extraction, enzyme-ultrasonic assisted extraction, enzyme extraction, and homogenate extraction (20). However, the products of SLPN or its extract are rarely, and utilization form are also need further research.

In this work, the environmentally friendly extraction method for nutritional and secondary metabolites from SLPN was optimized at room temperature, and the spray-drying process was used to manufacture an SLPN-instant beverage. The secondary metabolites, nutritional components, pesticides and heavy metal residues in SLPN and SLPN-instant beverage were analyzed by the vanillin-perchloric acid method, ultra-performance liquid chromatography coupled with electrospray time-of-flight mass spectrometry (UPLC-MS) and high performance liquid chromatography (HPLC). The antioxidant activity of the SLPN-instant beverage was determined *in vitro*. We provide a valuable process for the deep development and utilization of SLPN.

## Materials and methods

### Sample collection

In this study, three-year cultivation fresh SLPN was collected from the *Panax notoginseng* planting base of Gaotian Co. Ltd (104.27 E, 23.35 N, 1631 meters above sea level, Wenshan, China) in September 2021. Previous research indicates that vigorous ginsenosides transformation occurs in SLPN processed by sun-air drying and hot-air drying at 50^°^C, but not shade-air drying at 25^°^C (15). Therefore, in our experiments, SLPN were shade-air dried at 25^°^C for further experiments, and all samples are expressed as dry weight (DW).

### Chemicals reagents

Ginsenoside Re (G-Re), ginsenoside Rc (G-Rc), ginsenoside Rb_1_ (G-Rb_1_), ginsenoside Rb_2_ (G-Rb_2_), ginsenoside Rb_3_ (G-Rb_3_), ginsenoside Rg_1_ (G-Rg_1_), ginsenoside Rd (G-Rd), ginsenoside Rh_2_ (G-Rh_2_), notoginsenoside R_1_ (NG-R_1_), notoginsenoside R_2_ (NG-R_2_), notoginsenoside Fa (NG-Fa), notoginsenoside Fc (NG-Fc), notoginsenoside Fe (NG-Fe), notoginsenoside Fd (NG-Fd) and ginsenoside F_1_ (G-F_1_) were dissolved to 1.00 mg/ml as a standard solution with 70% (v/v) methanol, respectively. Ten polyphenols including catechin (C), gallic acid (GA), epicatechin (EC), 1,4,6-tri-O-galloyl-β-D-glucose (GG), epigallocahechin-3-gallate (EGCG), epicatechin-3-gallate (ECG), gallocatechin (GC), catechin gallate (CG), quercetin (Qc) and delphinidin (Dp) were prepared (1.00 mg/ml) as a standard solution in methanol, respectively. All the above chemicals were obtained from Chengdu Must Bio-technology Co. Ltd. (Chengdu, China), as reference standards (≥ 99% purity). Seventeen amino acid mixed standard reference with 0.1 nmol/μl were provided by Agilent Inc, including alanine (Ala), L-arginine (Arg), aspartic acid (Asp), cysteine (Cys), glutamic acid (Glu), glycine (Gly), histidine (His), isoleucine (Ile), leucine (Leu), serine (Ser), threonine (Thr), tyrosine (Tyr), valine (Val), methionine (Met), phenylalanine (Phi) and proline (Pro). Additionally, γ-aminobutyric acid (GABA) and dencichine (Dc) were dissolved in deionized water (1.00 mg/ml) as a standard solution. The commercial kits for *in vitro* antioxidant activity analysis, including scavenging activities (SCs) of 1,1-diphenyl-2-picrylhydrazyl (DPPH), 2,2′-azino-bis(3-ethylbenzothiazoline-6-sulfonate) (ABTS⋅^+^) radicals, hydroxyl (OH^–^) radicals and superoxide anion (O^2–^) radicals were purchased from Grace Biotechnology Co. Ltd. (Suzhou, China). Acetonitrile (ACN), methanol, and formic acid for HPLC analysis were purchased from MREDA (MREDA Technology Inc., Columbia, TN, USA). The deionized water was prepared using an ultrapure water on-line resistivity monitor (Shanghai, China).

### Optimization of extraction conditions

To give a high extraction rate and saponin content of SLPN, single factors, including ethanol concentration (v/v) (20–70%), material liquid ratio (m:V) (1:5–1:25) and extraction time (24, 36, 48, 60 and 72 h) were optimized. Then, a three-factor, three-level Box-Behnken design model (BBD) (Khatib et al. (21)) of ethanol concentration: –1 (30%), 0 (40%), +1 (50%), material liquid ratio (m:V): –1 (1:15), 0 (1:20), +1 (1:25) and extraction time: –1 (24 h), 0 (36 h), +1 (48 h) were examined using Design Expert 12.0.3.0 (Stat-Ease, USA) (Table 1). The design comprised 17 randomized runs with five replicates as the center points. The total saponin contents of SLPN-instant beverage were detected by vanillin-perchloric acid method (21), the extraction rate of SLPN in single factor experiment was calculated as Eq. 1:


(1) Singlefactorexperimentextractionrate(%)=(M-2M)1×100/[M×0(1-ω)]

**TABLE 1:** Designs and results of response surface experiments.

No.	*A*: Ethanol concentrations/%	*B*: Material liquid ratio/m:V	*C*: Extraction time/h	Extraction rate/%
1	0 (40%)	0 (1: 20)	0 (36 h)	41.60
2	+1 (50%)	0 (1: 20)	+1 (48 h)	37.22
3	–1 (30%)	+1 (1: 25)	0 (36 h)	34.80
4	0 (40%)	0 (1: 20)	0 (36 h)	41.48
5	–1 (30%)	–1 (1: 15)	0 (36 h)	32.04
6	+1 (50%)	+1 (1: 25)	0 (36 h)	37.44
7	0 (40%)	–1 (1: 15)	+1 (48 h)	36.03
8	0 (40%)	0 (1: 20)	0 (36 h)	39.09
9	0 (40%)	0 (1: 20)	0 (36 h)	38.10
10	0 (40%)	0 (1: 20)	0 (36 h)	40.18
11	0 (40%)	–1 (1: 15)	–1 (24 h)	34.93
12	–1 (30%)	0 (1: 20)	+1 (48 h)	31.49
13	+1 (50%)	0 (1: 20)	–1 (24 h)	34.41
14	–1 (30%)	0 (1:20)	–1 (24 h)	32.36
15	+1 (50%)	–1 (1: 15)	0 (36 h)	35.93
16	0 (40%)	+1 (1: 25)	–1 (24 h)	35.91
17	0 (40%)	+1 (1: 25)	+1 (48 h)	36.44

M_0_ is the mass of SLPN, unit (g); M_1_ is the mass of the evaporating dish, unit (g); M_2_ is the total mass of the evaporating dish and SLPN instant beverage, unit (g); ω is the water content of SLPN, unit (%), and the moisture (ω) of SLPN is 10.12 ± 0.17%.

### Manufacturing of stems and leaves *of Panax notoginseng*-instant beverage using stems and leaves of *Panax notoginseng*

A pilot-scale experiment was further carried out in a custom-designed tank reactor (50 L), and SLPN-instant beverage was manufactured as follows: one kilogram of SLPN was extracted by optimal methods, and the extraction solution was concentrated to 1/3 volume using a vacuum rotatory evaporator at 45^°^C, and then spray dried at 110^°^C. Then a powdered SLPN-instant beverage was obtained.

### Measurement of nutritional components

Seventeen amino acids were analyzed by HPLC which was reported in our previous paper (22) The method is given briefly below: 1 g SLPN powder was extracted with 50 ml distilled water for 2 h at 80^°^C and filtered through filter paper, the SLPN-instant beverage was dissolved in 50 ml distilled water. Then, 1 ml of SLPN extract and SLPN-instant beverage solution were mixed with 200 μl chloroform (CHCl_3_) and centrifuged at 12,000 g for 10 min at 4^°^C. Then 800 μl of each supernatant was filtered through a 0.2 μm nylon filter prior to the HPLC analysis.

The vitamins and mineral elements were determined by the Sanshu Co. Ltd. (Nanjing, China). The pesticide and heavy metal residues were evaluated by the Institute of Product Quality Supervision and Inspection, Yunnan, China. Fourteen pesticide residues, including hexachlorobenzene (BHC), dichloro-diphenyl-trichloroethane (DDT) were measured according to the People’s Republic of China national standard GB/T 5009.19; carbendazim and dimethomorph were measured according to the People’s Republic of China national standard GB/T 20769; procymidone, myclobutanil, propiconazole and oxadixyl were measured according to the People’s Republic of China national standard GB 23200; thiophanate-methyl was measured according to the People’s Republic of China national standard NY/T 1680; cyhalothrin was measured according to the People’s Republic of China national standard GB/T 5009.146. Heavy metal residues including As, Pb, Cd, and Hg were measured by Inductively Coupled Plasma Mass Spectrometer according to the People’s Republic of China national standards (GB 5009.11, GB 5009.12, GB 5009.15, GB 5009.17).

### Measurement saponin components

The amount of total saponin was determined by the vanillin-perchloric acid method, which is based on a color reaction of the acid-hydrolysis products of the saponin (i.e., sapogenins) with vanillin (21). One gram of SLPN powder was extracted with 50 ml of 70% (v/v) methanol for 1.5 h at 85^°^C. Subsequently, the extract solution was filtered to obtain the SLPN total saponin solution. One gram of SLPN-instant beverage was dissolved in 50 ml of 70% (v/v) methanol as a sample solution. Triple replicates of each SLPN and SLPN-instant beverage sample were extracted twice. A volume of 50 μl prepared sample solution was transferred into a colorimetric tube, then the solvent was volatilized at 60^°^C in a water bath. Freshly prepared 1.0% vanillin-perchloric acid solution reagent (0.5 ml) was added, mixed and incubated for 15 min at 60^°^C. Then, 5.0 ml 77% sulfuric acid (v/v) was added after immersion in an ice-water bath for 2 min. The absorbance was monitored using a spectrophotometer (Tu-190, Shanghai, China) at 535 nm using G-Re as a standard, while 70% (v/v) methanol and vanillin-perchloric acid was used as blank control. A standard curve of y=0.2229x− 0.0141 (*R*^2^ = 0.9979) with a linearity range of 0.5–8.5 mg/g was calculated, where y is the content of G-Re (mg) and *x* is the absorbance value. The results were expressed as mg/g-DW.

The principal secondary metabolites were subjected to qualitative analysis using UPLC-MS and HPLC system equipped with an evaporative light scattering detector (ELSD) (Waters, Milford, USA) at the Institute of Radiation Medicine, Academy of Military Medical Sciences (Beijing, China). The mobile phase flow rate was 0.35 ml/min with gradient elution of H_2_O + 0.1% formic acid (A) and acetonitrile (B) using the following gradient program: 0–3 min, 10–25% B; 3–8 min, 25–30% B; 8 - 13 min, 30–33% B; 13–15 min, 33–34% B; 15–17 min, 34–40% B; 17–19 min, 40–46% B; 19–21 min, 46–52% B; 21–23 min, 52–58% B; 23–25 min, 58–64% B; 25–26 min, 64–95% B; 26–27 min, 95% B; 27.5–29 min, 10% B. The column temperature and the detection wavelength were set 40^°^C and 203 nm, respectively. The MS conditions are below: Waters Vion IMS-Q-TOF system, ionization mode: electrospray ionization (ESI) ESI^–^ and ESI^+^, source temperature: 110^°^C, Desolvation temperature: 450^°^C, Desolvation gas flow: 850 L/h, capillary voltage: 2.5 kV (ESI^–^), 3.0 kV (ESI^+^), collision energy: low energy: 6 eV (ESI^–^), 6 eV (ESI^+^), high energy: 30–50 eV (ESI^–^), 40–65 eV (ESI^+^), collision gas: argon. The identification method of SLPN-instant beverage main components is mainly based on the molecular weight and error fragments of the UNIFI database as well as the reference related analytical literature. Identification of ginsenoside and polyphenol compounds were dependent on the primary and secondary MS data, annotated against an in-house database, or the matches of both retention times and MS data to some standard compounds in the UNIFI database.

Monomer saponins were quantitative analyzed by high-performance liquid chromatography (HPLC-1290 identify II) system equipped with a diode array detector (DAD), quaternary pump, column compartment and autosampler. A Poroshell EC-C_18_ chromatographic column (4.6 × 150 mm, 4 μm) (Agilent, USA) was adopted for the analyses. A gradient elution system consisted of ultra-pure water (A) and acetonitrile (B) using the following gradient program: 0–20 min, 20% B; 20–55 min, 20–36% B; 55–70 min, 36–45% B; 70–79 min, 45–60% B; 79–80 min, 80% B; 80–81 min, 80% B; 81.5–83 min, 20% B. The flow rate was set at 0.5 ml/min and the sample volume was set at 10 μl. The column temperature and the detection wavelength were set 30^°^C and 203 nm, respectively.

### Measurement polyphenols components

Polyphenols was measured on an HPLC system (Agilent 1200) according to the method described in our previous paper (23). The HPLC was equipped with a variable wavelength detector (VWD) and Poroshell 120 EC-C_18_ chromatographic column (4.6 × 100 mm, 2.7 μm, Agilent, USA). SLPN polyphenol extract preparation was as follows: SLPN samples were ground to a fine powder and passed through a 40-mesh sieve. SLPN sample (1 g) was extracted with 44 ml MeOH-hydrochloric acid (40:4 v/v) in a flask equipped with a reflux condenser, the extraction was performed for 90 min in an 85^°^C water bath. Then, SLPN polyphenol extract and SLPN-instant beverage were diluted to 50 ml. The diluted solutions were then filtered through a 0.45 μm nylon filter and immediately analyzed by HPLC. The optimal elution conditions were as follows: A (ultra-pure water + 5% ACN + 0.261% ortho-phosphoric acid), B (80% MeOH); 0–16 min, 10–45% B; 16–22 min, 45–65% B; 22–25.9 min, 100% B; 25.9–29.9 min, held at 100% B. The flow rate was 0.8 ml/min, and each sample was detected in triplicate.

### Antioxidation assays

The antioxidant activities of SLPN and SLPN-instant beverage were investigated *in vitro*, the methods were referenced by Wang et al reports (24). The antioxidant activity SLPN and SLPN-instant beverage were evaluated using the following assays: scavenging activities (SC) of DPPH, ABTS⋅^+^, OH^–^, and O^2–^ radicals, Trolox solution was used as the positive control. These activities were determined using commercial kits (Grace Biotechnology Co. Ltd., Suzhou, China) according to the manufacturer’s instructions.

### Statistical analysis

Data analysis was performed in SPSS 26.0 (IBM, New York, USA) and the results are expressed as mean ± standard deviation (SD). Single factors experiment and antioxidant activity results were analyzed and plotted with GraphPad prism 8.3.0 (GraphPad Prism, San Diego, California, USA). Response surface design and analysis of variance (ANOVA) were performed by Design Expert 12.0 software. The heat maps of the chemical components were plotted by TB-tools v 0.068 (25). Three replicates of each sample were extracted, and each extraction was analyzed twice. *P* < 0.05 was considered to be statistically significant.

## Results and discussion

### Optimization of the extraction

**FIGURE 1:**
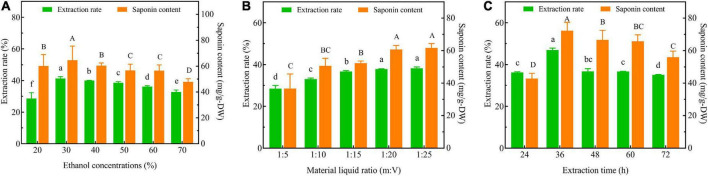
Extraction rate and total saponin content of SLPN-instant beverage optimized by single factor test. Extraction rate (%) as a function of **(A)** ethanol concentration (%), **(B)** material liquid ratio (m:V) and **(C)** extraction time (h) (Different letters indicate significant differences based on ANOVA *post-hoc* test at *P* < 0.05).

**FIGURE 2:**
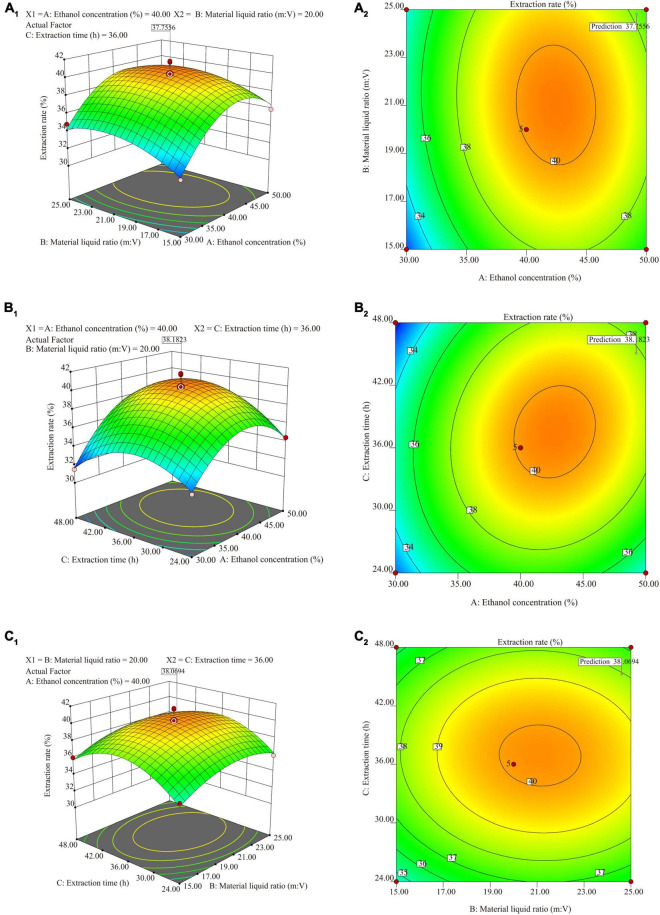
The three-dimensional curved surface graph and contour plots for ethanol concentration vs. material liquid ratio (m:V) **(A_**1**_,A_**2**_)**; ethanol concentration vs. extraction time **(B_**1**_,B_**2**_)**; material liquid ratio vs. extraction time **(C_**1**_,C_**2**_)**.

Three-factor single experiments were used to optimize the extraction process. The extraction rates and total saponin contents of SLPN at 30% ethanol concentration (v/v) (Figure 1A), 1:20 material liquid ratios (m:V) (Figure 1B) and 36 h extraction time (Figure 1C) were significantly higher than the ones at other extract conditions, respectively (*P* < 0.05). The BBD model of ethanol concentrations *A* (30, 40, and 50%), material liquid ratio *B* (1:15, 1:20, 1:25) and extraction time *C* (24, 36 and 48 h) were further developed to optimize the extraction conditions, and consequently the extraction rate was increased from 31.49 to 41.60% (Table 1). According to the ANOVA analysis as shown in Table 2, alcohol concentration (*A*), secondary terms *A*^2^ and *C*^2^ showed highly significant effects on the extraction rate (*P* < 0.01), and the secondary term *B*^2^ also had a significant effect on the extraction rate (*P* < 0.05). In “lack of fit” analysis an *F*-value = 0.9043 and *P*-value = 0.18 indicates that the quadratic model data are suitable for representing the whole experimental data. The three-dimensional response surface plots and corresponding two-dimensional contour plots constructed by *AB* showed that the extraction rate of SLPN-instant beverage was increased when the ethanol concentration (*A*) increased from 20 to 48%, material liquid ratio (*B*) increased from 1:5 to 1:20. While the extraction rate decreased with the further increase in the two factors (Figures 2A_1_,A_2_). For the interaction between the ethanol concentration (*A*) and the extraction time (*C*), the extraction rate of SLPN-instant beverage was increased along with the extraction time increasing from 12 to 38 h. However, it decreased if the extraction time was continually extended (Figures 2B_1_,B_2_). Moreover, the extraction rate was highest when the material liquid ratio (*B*) approached 1:20 and the extraction time (*C*) was extended to 38 h (Figures 2C_1_,C_2_). These results indicated that the extraction rates were extremely affected by the ethanol concentration, material liquid ratio, and extraction time. Consequently, the regression equation of the extraction rate is fitted as below (Eq. 2).


(2) $${\text{Y}}_{\mathrm{Extaction}\,\mathrm{rate}}\left(\%\right)=40.09+1.79\text{A}+0.7075\text{B}+0.4463\text{C}-0.3125\,\text{AB}+0.92\,\text{AC}-0.1425\,\text{BC}-3.5\,{\text{A}}^{2}-1.54\,{\text{B}}^{2}-2.72\,{\text{C}}^{2}$$

**TABLE 2:** Variance analysis of the regression model.

Source	Sum of squares	*df*	Mean square	*F*-value	*P*-value	Significant
Model	137.29	9	15.25	10.24	0.0029	**
*A* - Ethanol concentrations (%)	25.60	1	25.60	17.19	0.0043	**
*B* - material liquid ratio (m: V)	4.00	1	4.00	2.69	0.1451	
*C* - Extraction time (h)	1.59	1	1.59	1.07	0.3354	
*AB*	0.39	1	0.39	0.26	0.6243	
*AC*	3.39	1	3.39	2.27	0.1753	
*BC*	0.08	1	0.08	0.05	0.8220	
*A* ^2^	51.51	1	51.51	34.59	0.0006	**
*B* ^2^	9.99	1	9.99	6.71	0.0360	*
*C* ^2^	31.21	1	31.21	20.96	0.0026	**
Residual	10.42	7	1.49			
Lack of Fit	1.24	3	0.41	0.18	0.9043	not significant
Pure Error	9.18	4	2.30			
Cor Total	147.71	16				
*R*^2^ = 0.93	*R*^2^*_(Adj)_* = 0.84		*R*^2^*_(Pre)_* = 0.77		*C.V*.% = 3.35%	
Adeq Pre = 9.06						

”*” means significant at *P* < 0.05, “**” means extremely significant at *P* < 0.01.

**FIGURE 3:**
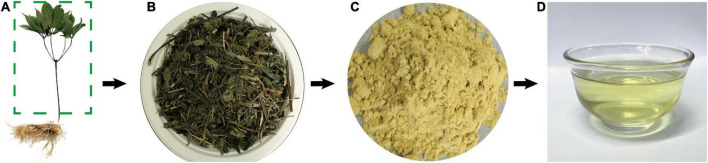
The manufacturing process of SLPN-instant beverage. **(A)** Fresh SLPN material; **(B)** SLPN material dried at 25^°^C shade-air; **(C)** SLPN-instant beverage; **(D)** infusion of SLPN-instant beverage.

Based on the desirability of reducing the sample consumption and reagents in the experiment, the optimal factors were obtained according to the BBD model while taking economic development and environmental protection requirements into consideration. The BBD model suggests that the maximum value of the extraction rate might reach 39.28% when the ethanol concentration (*A*) is 48.42% (w/v), material liquid ratio (*B*) is 1:20.64 (m:V) and extraction time (*C*) is 38.58 h, respectively. Triplicate experiments were used to verify the model’s reliability and accuracy, and consequently the extraction rate was 37.89 ± 0.02%. To further investigate the practical availability in industrial application, one kilogram of SLPN was extracted by the above-suggested method, and consequently 361.48 ± 4.57 g of SLPN-instant beverage was obtained, and the extraction yield was 36.15 ± 0.46%, being in good agreement with the predicted values. Organoleptic evaluation results showed that the SLPN-instant beverage was a fine powder with a yellow-green color (Figures 3A–C), and it showed good solubility in both cold and hot water (Figure 3D), and the taste of the solution was slightly bitter with a sweet aftertaste.

We analyzed the pesticide and heavy metals residues in SLPN and SLPN-instant beverage, the results showed that the content of lead (Pb), cadmium (Cd), carbendazim, procymidone, and dimethomorph were 2.20 mg/kg, 0.94 mg/kg, 140 mg/kg, 4.48 mg/kg and 42.8 mg/kg in SLPN, respectively, were higher than the DBS 53 024–2017 standard limits (26). Interestingly, these contaminants were 0.11, 0.08 mg/kg, 0.47 mg/kg, 0.03 mg/kg and 0.19 mg/kg in the SLPN-instant beverage, respectively (Table 3), all being below the DBS 53 024–2017 limits. These results showed that our extraction method could reduce the pesticide and heavy metal residues in the instant beverage.

**TABLE 3:** Pesticide and heavy metal residues in SLPN and SLPN-instant beverage.

No.	Items	Standard limit (mg/kg)	SLPN (mg/kg)	SLPN-instant beverage (mg/kg)
1	Total Pb	≤ 2.00	2.20*	0.11
2	Total As	≤ 1.50	1.34	0.24
3	Total Cd	≤ 0.50	0.94*	0.08
4	Total Hg	≤ 0.10	0.03	<0.01
5	Benzene hexachloride (BHC)	≤0.05	N.d	N. d
6	Dichloro-diphenyl-trichloroethane (DDT)	≤ 0.05	N.d	N. d
7	Carbendazim	≤ 5.00	140*	0.47
8	Procymidone	≤ 4.00	4.48*	0.03
9	Thiophanate-methyl	≤ 3.00	0.50	N. d
10	Myclobutanil	≤ 1.00	N.d	N. d
11	Dimethomorph	≤ 2.00	42.8*	0.19
12	Propiconazole	≤ 0.50	N.d	N. d
13	Cyhalothrin	≤ 0.20	N.d	N. d
14	Oxadixyl	≤ 1.00	N.d	N. d

“*” Means items over the limits of local standards DBS 53 024 - 2017 dried stems and leaves of *panax notoginseng*, N.d means not detected (*n* = 3).

### Nutritional components in stems and leaves of *Panax notoginseng* and stems and leaves of *Panax notoginseng*-instant beverage

**FIGURE 4:**
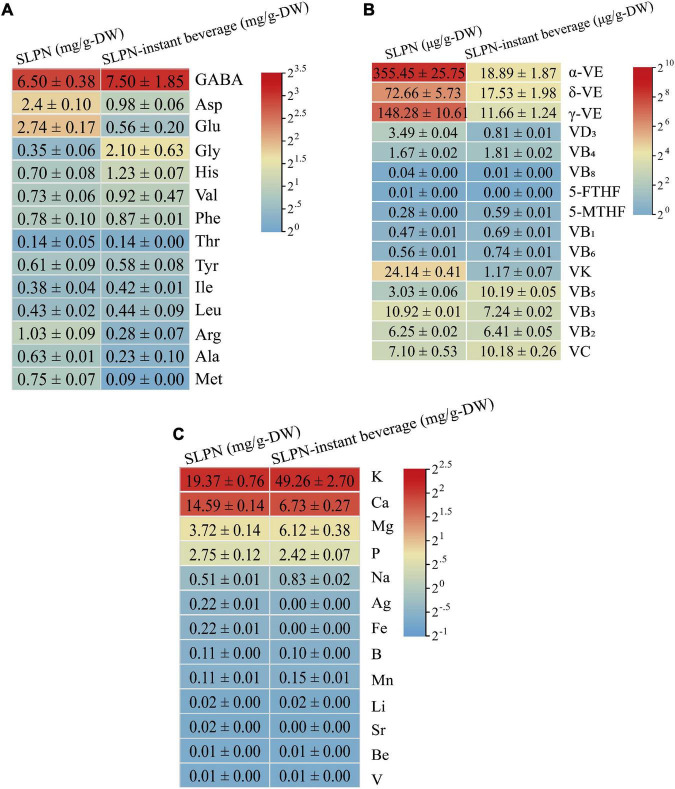
The contents of **(A)** fourteen amino acids **(B)** fifteen vitamins and **(C)** thirteen mineral elements in SLPN and SLPN-instant beverage.

The nutritional components in SLPN and SLPN-instant beverage including amino acids, vitamins, and mineral elements were also analyzed, respectively. Fourteen amino acids were detected in SLPN and SLPN-instant beverage (Figure 4A). In SLPN, the contents of γ-aminobutyric acid were 6.5 ± 0.38 mg/g-DW, Asp 2.40 ± 0.10 mg/g-DW, Glu 2.74 ± 0.17 mg/g-DW and Gly 0.35 ± 0.06 mg/g-DW, whereas in SLPN-instant beverage, the contents of γ-aminobutyric acid were 7.50 ± 1.85 mg/g-DW, Gly 2.10 ± 0.63 mg/g-DW and His 1.23 ± 0.07 mg/g-DW, respectively. Additionally, ten mineral elements were determined, and potassium 49.26 ± 2.70 mg/g-DW, magnesium 6.12 ± 0.38 mg/g-DW and sodium 0.83 ± 0.02 mg/g-DW were enriched in SLPN-instant beverage, but calcium 6.73 ± 0.27 mg/g-DW and phosphorus 2.42 ± 0.07 mg/g-DW were relatively decreased (Figure 4B). Fifteen vitamins, including vitamin B, vitamin C, vitamin D, vitamin E were detected in SLPN. The contents of different monomer vitamins ranged from 0.01 μg/g-DW to 355.45 μg/g-DW, and the main components were α-VE 355.45 ± 25.75 μg/g-DW, γ-VE 148.28 ± 10.61 μg/g-DW, δ-VE 72.66 ± 5.73 μg/g-DW and VK 24.14 ± 0.41 μg/g-DW. Meanwhile, the contents of different monomer vitamins ranged from 0 to 18.89 μg/g-DW in SLPN-instant beverage and the main components were α-VE 18.89 ± 1.87 μg/g-DW, γ-VE 11.66 ± 1.24 μg/g-DW, δ-VE 17.53 ± 1.98 μg/g-DW, and VB5 10.19 ± 005 μg/g-DW, respectively (Figure 4C).

### Secondary metabolites in in stems and leaves of *Panax notoginseng* and in stems and leaves of *Panax notoginseng*-instant beverage

**FIGURE 5:**
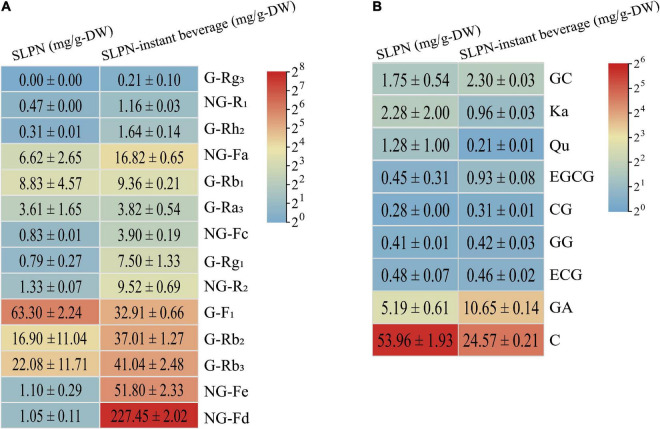
The contents of **(A)** fourteen saponin and **(B)** nine polyphenols in SLPN and SLPN-instant beverage.

The result of UV spectrophotometry showed that the total saponin in SLPN was 100.19 ± 10.08 mg/g-DW, while it reached 403.05 ± 34.98 mg/g-DW in SLPN-instant beverage. The content of total saponin in SLPN-instant beverage was increased about quadruple than that of SLPN raw material. Forty-three triterpenoid ginsenosides (e.g., ginsenoside La, G-Rb_3_, notoginsenoside D, NG-R_1_), and two flavonoid glycoside including quercetin-3-O-β-D-galactose (2→1) glucoside and kaempferol-3-O-β-D-galactose (2→1) glucoside, as well as dencichine were identified in SLPN by UPLC-MS, as showed in Supplementary Table 1 and Supplementary Figure 2. Additionally, the secondary metabolites of SLPN-instant beverage were tentatively identified by HPLC-ELSD, the results were shown in Table 4 and Supplementary Figure 2B. In our experiments, fourteen monomer ginsenosides were quantitatively analyzed by HPLC, the content of each monomer ginsenoside was between 0 and 63.3 mg/g-DW in SLPN, and the main ginsenosides were G-F_1_ 63.30 ± 2.24 mg/g-DW, G-Rb_3_ 22.08 ± 11.71 mg/g-DW, G-Rb_2_ 16.9 ± 11.04 mg/g-DW, and G-Rb_1_ 8.83 ± 4.57 mg/g-DW, respectively. The more abundant ginsenosides in the SLPN-instant beverage were NG-Fd 227.45 ± 2.02 mg/g-DW, NG-Fe 51.80 ± 2.33 mg/g-DW, G-Rb_3_ 41.04 ± 2.48 mg/g-DW, and G-Rh_2_ 1.64 ± 0.14 mg/g-DW (Figure 5A).

**TABLE 4:** Principal secondary metabolites in SLPN-instant beverage identified by HPLC-ELSD.

No.	Retention time (min)	Tentative identification	Formula	[M-H]^–^
				
1	2.84	Quercetin-3-o-β-D-galactose (2→1) glucoside	C_27_H_30_O_17_	625.1461
2	3.18	Kaempferol-3-o-β-D-galactose (2→1) glucoside	C_27_H_30_O_16_	609.1522
3	11.81	Notoginsenoside Fa	C_59_H_100_O_27_	1239.6357
4	12.87	Notoginsenoside Fc/FP2	C_63_H_106_O_31_	1209.6383
5	14.39	Notoginsenoside Fc/FP2	C_63_H_106_O_31_	1209.6383
6	15.01	Malnoylfloralginsenosides Rc_1/2/3/4_	C_56_H_92_O_25_	1163.5974
7	16.54	Malnoylfloralginsenosides Rc_1/2/3/4_	C_56_H_92_O_25_	1163.5974
8	18.43	Notoginsenoside K	C_48_H_82_O_18_	945.5522
9	19.09	Gypenoside IX/Notoginsenoside Fe/Notoginsenoside Ft1	C_47_H_80_O_17_	915.5405
10	19.47	Gypenoside IX/Notoginsenoside Fe/Notoginsenoside Ft1	C_47_H_80_O_17_	915.5405
11	19.66	Gypenoside IX/Notoginsenoside Fe/Notoginsenoside Ft1	C_47_H_80_O_17_	915.5975

Additionally, nine phenolic compounds, including catechin, GA, GC, CG etc., were detected in SLPN and its beverage (Figure 5B). The content of total polyphenol did not show a significant difference between SLPN and SLPN-instant beverage. In SLPN, the main phenolic compounds were catechin 53.96 ± 1.93 mg/g-DW, GA 5.19 ± 0.61 mg/g-DW, Ka 2.28 ± 2.00 mg/g-DW, GC 1.75 ± 0.54 mg/g-DW, and Qu 1.28 ± 1.00 mg/g-DW. After extraction, the contents of catechin 24.57 ± 0.21 mg/g-DW, ECG 0.46 ± 0.02 mg/g-DW, Qu 0.21 ± 0.01 mg/g-DW and Ka 0.96 ± 0.03 mg/g-DW were decreased in SLPN-instant beverage. While the level of GA 10.65 ± 0.14 mg/g-DW was increased in the SLPN-instant beverage.

### Saponins and polyphenols affect the antioxidant activity of in stems and leaves of *Panax notoginseng*-instant beverage

**FIGURE 6:**
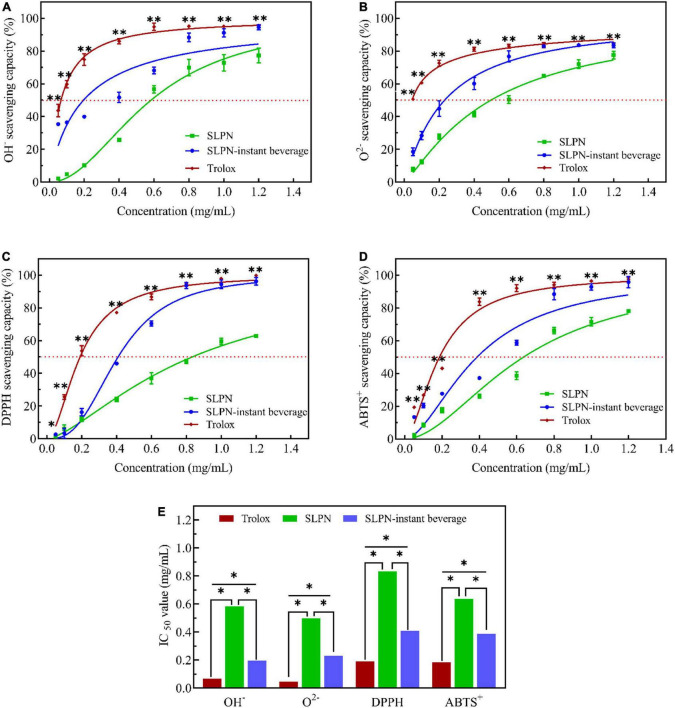
Scavenging activities of OH^–^ radicals **(A)**, O^2–^ radicals **(B)**, DPPH radicals **(C)**, ABTS^+^ radicals **(D)**, and IC_50_ **(E)** in SLPN and SLPN-instant beverage, Trolox as positive control. *Indicates a significant difference, **Indicates a very significant difference based on ANOVA *post-hoc* test (including *t*-test) at *P* < 0.05.

According to the optimized extraction method, nutrients and secondary metabolites of SLPN are enriched in this experiment. SLPN-instant beverage is rich in nutrients and secondary metabolites, including saponins (G-Rb_1_, G-Rb_3_, NG-Fd), polyphenols (C, GA, GC), amino acids (Asp, Glu, Gly), and multivitamin etc. *In vitro* assays, SLPN-instant beverage showed good scavenging activities (SCs) for hydroxyl radicals (Figure 6A), superoxide anion radicals (Figure 6B), DPPH (Figure 6C), and ABTS⋅^+^ (Figure 6D) with dose-dependent behavior. The IC_50_ for SCs of OH^–^ radicals, O^2–^ radicals, DPPH radicals and ABTS^+^ radicals were 0.5826, 0.4966, 0.8331, and 0.6368 mg/ml, respectively (Figure 6E). While it was 0.1954, 0.2314, 0.4083, and 0.3874 mg/ml for SLPN-instant beverage to scavenge these radicals (Figure 6E). Previous reports indicated that Gypenoside IX (NG-Fd) isolated from SLPN significantly suppressed the nitric oxide production and inflammatory cytokines including tumor necrosis factor-α, interleukin 10, interferon-inducible protein 10 and interleukin-1β, shows good antioxidant stress and anti-inflammatory effects (27), ginsenoside Rb_3_ could decrease oxidative stress and protect endothelial function in hypertension (28).

## Discussion

### A single factor test and a response surface design improve the extraction rate of stems and leaves of *Panax notoginseng* -instant beverage

The extraction process could be optimized for maximizing the contents of functional components from ginger using a single factor test and a response surface BBD design (29). In a single factor test experiment, when the ethanol concentration was increased from 20 to 30%, the extraction rate was increased from 28.65 ± 3.77% to 41.26 ± 1.13% (Figure 1A). According to the principle of liquid-liquid mass transfer (30), the soluble components separate out from the extraction process, but it might be adsorbed on the SLPN interface with the increase in ethanol concentration that reduces the total mass transfer coefficient and the extraction rate of SLPN-instant beverage. The extraction rate of SLPN-instant beverage increases with the increase of the material liquid ratio (m:V). When the material liquid ratio was increased from 1:20 to 1:25, the extraction rate was increased from 37.82% ± 0.15% to 38.23% ± 0.64% (Figure 1B), indicating that the material liquid ratio 1:20 is suitable for the extraction process. In our study, when the extraction time exceeded 36 h, the extraction rate decreased rapidly. The solvent-terminated dispersive liquid-liquid microextraction model (31) has explained that with the prolonged extraction time, the ethanol extractant either floats or sinks depending on the density of aqueous SLPN as has been observed in our study (Figure 1C). Therefore, the bioextraction technology is a complex process, a single factor cannot completely investigate the best extraction process of SLPN-instant beverages, since it involves interacting multiple extraction factors (32).

In the response surface design experiment, when the ethanol concentration (*A*) was 30–50%, material liquid ratio (*B*) 1:15–1:20, extraction time (*C*) 36 h, the highest point of the extraction rate in 3D hook face reached 37.76% (Figures 2A_1_,A_2_). As shown in Table 2, low *P*-values (*P* < 0.01 and *P* < 0.5) of BBD model associated with high *F*-values (10.24 and 17.19) implied the significance of the model (33). The extraction condition of the present study is similar to that of the other study that polyphenols are extracted from *P. quinquefolius* L., and the extraction rate reached 48% (34). The interaction between (*A*) and (*C*) showed that when the ethanol concentration is 30–40%, material liquid ratio 1:20, extracted time 24–36 h, the highest extraction rate reaches 38.18% (Figures 2B_1_,B_2_). When the material liquid ratio was 1:15–1:20, extracted time 24–36 h, with 40% of ethanol concentration, the highest extraction rate reached 38.18% (Figures 2C_1_,C_2_). Consequently, the BBD model recommends that the optimal condition is 48.42% ethanol concentration, material liquid ratio 1:20.64, and extraction time 38.58 h. To facilitate the experimental operation, we have made a minor adjustment, that is, 48.50% of ethanol concentration, material to liquid 1:21, extracted time 39 h, and repeated twice. The extraction rate under the interaction of the three factors can reach 37.89 ± 0.02%. In our previous work, the extraction yield of *P. notoginseng* flowers is 46.63 ± 0.81%, being higher than the extract yield of SLPN-instant beverage based on the adjusted method. Furthermore, a pilot-scale experiment was further carried out in a custom-designed tank reactor (50 L), one kilogram of SLPN was extracted by the adjusted method, and consequently 361.48 ± 4.57 g-DW of SLPN-instant beverage was obtained, and the extraction yield was 36.15 ± 0.46% (unpublished data). Thus, our experiment optimized a stable extraction method from a response surface model to manufacture SLPN-instant beverage (Figures 3A–D).

### Nutritional components of in stems and leaves of *Panax notoginseng* and in stems and leaves of *Panax notoginseng*-instant beverage

It has been reported that SLPN contain amino acids, vitamins, polysaccharides, and other nutrients (9, 35). The total content of eighteen free amino acids in SLPN is 0.344% (36). In our experiment, fourteen key amino acids in SLPN and SLPN-instant beverage were detected by HPLC, and the main amino acids in SLPN-instant beverage were γ-aminobutyric acid, Gly, Asp, Val and His (Figure 4A). In many plant-derived foods, γ-aminobutyric acid acts as a main inhibitory neurotransmitter in the central nervous system (37). Thus, SLPN and SLPN-instant beverage might improve anti-fatigue and extend the sleeping function (38). Vitamins are one of the important parts in food nutrition, and in our study fifteen vitamins were detected in SLPN and SLPN-instant beverage. Unfortunately, the main components in SLPN-instant beverage such as α-VE, γ-VE, δ-VE, and VB had not been enriched in our experiments (Figure 4B). Ten mineral elements including potassium, magnesium and sodium were enriched in the SLPN-instant beverage (Figure 4C). We found that the content of pesticides and heavy metal residues in the SLPN-instant beverage conformed to the limit of the food standard DBS 53 024–2017. Therefore, SLPN-instant beverage is safe and edible, and it can be used as one of the supplementary of nutrition sources for human body.

### Secondary metabolites of aqueous extract of in stems and leaves of *Panax notoginseng* and in stems and leaves of *Panax notoginseng*-instant beverage

Saponin is the most important secondary metabolites in plants of the genus *Panax* (39). In our experiment, the total saponin of SLPN was 100.19 ± 10.08 mg/g-DW, within the reported scope of saponin content ranging from 60 to 100 mg/g-DW (40, 41). While the total saponin content of SLPN-instant beverage was 403.05 ± 34.98 mg/g-DW, it increased nearly four times as compared with that of SLPN. Total saponin of SLPN show multiple pharmacological activities, e.g., cardioprotective (42), hepatoprotective (43), anti-inflammatory (8) and inhibition of depression effects (44). The total saponin of SLPN could activate the PI3K/Akt/mTOR signaling pathway to attenuate excessive autophagy and apoptosis in myocardial cells in heart tissue induced by sleep deprivation (42). Furthermore, fermented *P. notoginseng* leaves could increase the content of secondary metabolites, and show a good antifatigue effect in vivo and *in vitro* (45), and the ethanol-induced metabolic perturbations are restored when treated by SLPN saponin (42). In our experiment, forty-three triterpenoid ginsenosides were identified in SLPN by UPLC-MS (Supplementary Table 1, Supplementary Figure 2A). G-Rb_3_, G-Rc, G-Rb_2_, G-Rb_1_, and NG-Fd have been regarded as the most important ginsenoside compounds in *P. notoginseng* leaves (46). While, in the SLPN-instant beverage, the principal components were NG-Fa, notoginsenoside-Fc/FP_2_, malonylfloralginsenosides Rc_1/2/3/4_, and gypenoside IX (GP-IX)/notoginsenoside Fe/notoginsenoside Ft_1_ (Table 4, Supplementary Figure 2B).

The most abundant secondary metabolite was NG-Fd at 1.05 ± 0.11 mg/g-DW, 227.45 ± 2.02 mg/g-DW in SLPN and SLPN-instant beverage, respectively (Figure 5A). Recent research has shown that 30 mg of refined SLPN extract could produce 8.4 mg of NG-Fd with a purity of 87.3% (47). NG-Fd might suppress reactive astrogliosis to treat neuroinflammatory disorders (48) and show a moderate anti-inflammatory effect (8). Therefore, NG-Fd could be served as an important pharmacological active saponin in the SLPN. Although the content of NG-Fe was 1.10 ± 0.29 mg/g-DW in SLPN, it was 51.80 ± 2.33 mg/g-DW in the SLPN-instant beverage (Figure 5A). NG-Fe could decrease food intake and body weight, protect liver structure integrity and normal function, as well as promote a resting metabolic rate in C57BL/6 mice (49). Also, NG-Fe is one of the key components to improve chronic obstructive pulmonary disease (50). G-Rb_3_ is one of the most important components in the SLPN and its content is 6.66–29.85 mg/g (51). In our experiment, 22.08 ± 11.71 mg/g-DW and 41.04 ± 2.48 mg/g-DW of G-Rb_3_ was detected in SLPN and the SLPN-instant beverage, respectively (Figure 5A). G-Rb_3_ might inhibit pro-inflammatory cytokines (52), suppress myocardial fibrosis (53), provide protective effects against cisplatin-induced nephrotoxicity (54), decrease oxidative stress and protect endothelial function in hypertension (28) and alleviate smoke-induced lung injury (55). Furthermore, in our study the content of minor ginsenoside Rh_2_ in the SLPN-instant beverage was 1.64 ± 0.14 mg/g-DW, being five times higher than that in SLPN. G-Rh_2_ could inhibit colorectal cancer cell growth (56), suppress the proliferation of A549 cells (57), breast cancer cell growth (58), and moreover the combination of G-Rh_2_ could induce lung cancer (59). Up to date, G-Rh_2_ is being developed as a new antitumor drug. Overall, SLPN-instant beverage might qualify a potential application in functional foods as it is rich in total saponin and polyphenols.

### Antioxidation of in stems and leaves of *Panax notoginseng* and in stems and leaves of *Panax notoginseng*-instant beverage

Medicinal plants *P. notoginseng* and SLPN are rich in secondary metabolites, especially saponins and polyphenols. In our experimental results, the SLPN-instant beverage is rich in G-Rb_1_, G-Rb_3_, NG-Fd, catechin, gallic acid, gallocatechin, multiple amino acids (Asp, Glu, Gly) and vitamins. SLPN flavonoid extract reveals a good antioxidant potential *in vitro* assay, the IC_50_ for O^2–^ at 2.89 mg/ml, DPPH at 7.21 mg/ml and ABTS^+^ at 2.95 mg/ml (60). In our study, the IC_50_ for SLPN to OH^–^ radicals was at 0.5826 mg/mL, O^2–^ radicals at 0.4966 mg/mL, DPPH radicals at 0.8331 mg/mL and ABTS^+^ radicals at 0.6368 mg/mL, respectively. While the IC_50_ for SLPN-instant beverage to scavenge these radicals was 0.1954 mg/mL, 0.2314 mg/mL, 0.4083 mg/mL, 0.3874 mg/mL (Figure 6E). Thus, the antioxidant activity of the SLPN-instant beverage is better than that of SLPN aqueous extract. Saponins and polyphenols have been largely used in various pharmaceutical and food industries (61). It has been demonstrated that 5 μg/mL of *P. notoginseng* leaves total saponin could reduce cell death induced by H_2_O_2_ (62). Therefore, the SLPN-instant beverage manufactured from SLPN is rich in total saponins, polyphenols, ginsenosides and nutrition, shows a good antioxidation and might be neuroprotective in neurological disorders. Previous reports indicated that fermented SLPN increased liver glycogen and serum lactate dehydrogenase activity, decreased blood urea nitrogen, lactate acid, and malondialdehyde in mice, showed a good antifatigue effect and *in vivo* (45). SLPN saponins could inhibit abnormal autophagy and produce cardioprotective effects in mice (42). Thus, in future studies, we will take the main function of the SLPN-instant beverage as a research goal, and deeply explore the key target *in vivo*.

## Conclusion

A SLPN-instant beverage has been manufactured from the food resources SLPN according to a single factor test and a response surface design, and the extraction rate reach 37.89 ± 0.02%. The total saponin in the SLPN-instant beverage is four times greater than that in SLPN. The SLPN-instant beverage is rich in ginsenosides, polyphenols, amino acids, vitamins, and mineral elements, and it shows a good antioxidant activity *in vitro*. Our experiments provide a simple, reliable, low cost and environmentally friendly technology to improve the utilization rate of the medicinal crops of *P. notoginseng*.

## Data availability statement

The original contributions presented in this study are included in the article/Supplementary material, further inquiries can be directed to the corresponding authors.

## Author contributions

JC and MZ: conceiving and designing the project. ZL and KL: analyzing the data and writing the manuscript. BM: identification of chemical composition. GZ: funding acquisition. SY and YZ: providing laboratory platform. All authors contributed to the article and approved the submitted version.
