# Supplementary material for: An instant beverage rich in nutrients and secondary metabolites manufactured from stems and leaves of Panax notoginseng
Source: Front Nutr. 2022 Dec 7;9:1058639. doi: 10.3389/fnut.2022.1058639 (PMC9767984; doi:10.3389/fnut.2022.1058639)
Supplement: Supplementary file 2 [file Table_2.DOCX]

**Supplementary information**

**Appendix. Supplementary data**

**Table S1.** Components identified in SLPN by LC-MS.

**Figure S1.** Photograph of *Panax notoginseng* plants.

**Figure S2.** Base peak ion chromatogram (BPI) of SLPN analyzed by UPLC - MS (A), principal components of instant beverage identified by HPLC-ELSD (B).

**Table S1.** Components identified in SLPN by LC-MS.

| No. | Component | Retention time (min) | Formula | Observed (*m/z*) | Mass error  (m Da) | Mass error  (ppm) | Adducts |
| --- | --- | --- | --- | --- | --- | --- | --- |
| 1 | Dencichine | 0.66 | C_5_H_8_N_2_O_5_ | 175.0357 | -0.3 | -1.7 | -H |
| 2 | Quercetin-3-O-β-D-galactose (2⭢1) glucoside | 2.84 | C_27_H_30_O_17_ | 625.1461 | -1.4 | -0.8 | -H |
| 3 | Kaempferol-3-O-β-D-galactose (2⭢1) glucoside | 3.18 | C_27_H_30_O_16_ | 609.1522 | -1.3 | -1.1 | -H |
| 4 | Ginsenoside-La | 7.2 | C_42_H_72_O_14_ | 845.4891 | -1.3 | -1.5 | +HCOO |
| 5 | Floralginsenoside P | 9.63 | C_53_H_90_O_23_ | 1139.5840 | -1.6 | -1.4 | +HCOO, -H |
| 6 | Notoginsenoside D | 10.66 | C_64_H_108_O_31_ | 1417.6830 | -3 | -2.1 | +HCOO, -H |
| 7 | Ginsenoside I (24S or 24R) | 11.06 | C_48_H_82_O_20_ | 977.5307 | -1.9 | -2 | -H |
| 8 | Chikusetsusaponin L_5_ | 11.72 | C_46_H_78_O_17_ | 947.5195 | -2.6 | -2.8 | +HCOO |
| 9 | Notoginsenoside R_4_ | 12.16 | C_59_H_100_O_27_ | 1285.6410 | -2.8 | -2.2 | +HCOO, -H |
| 10 | Notoginsenoside R_1_ | 12.54 | C_47_H_80_O_18_ | 977.5298 | -2.9 | -2.9 | +HCOO |
| 11 | Floralquinquenoside B | 12.55 | C_42_H_72_O_15_ | 815.4780 | -1.8 | -2.2 | -H |
| 12 | Ginsenoside F_3_ | 12.81 | C_41_H_70_O_13_ | 815.4786 | -1.3 | -1.5 | +HCOO |
| 13 | Ginsenoside Ra_1_ | 13.35 | C_58_H_98_O_26_ | 1255.6290 | -3.4 | -2.7 | +HCOO, -H |
| 14 | Malonylginsenoside Ra_3_ | 13.7 | C_62_H_102_O_30_ | 1325.6350 | -3.0 | -2.3 | -H |
| 15 | M-notoginsenoside R_4_ | 13.9 | C_62_H_102_O_30_ | 1325.6350 | -3.3 | -2.5 | -H |
| 16 | Yesanchinoside J | 14.15 | C_61_H_102_O_28_ | 1281.6450 | -3.4 | -2.6 | -H |
| 17 | Quinquefoloside-Lc | 14.28 | C_54_H_92_O_23_ | 1153.5980 | -3 | -2.6 | +HCOO, -H |
| 18 | Quinquenoside R_1_ | 14.57 | C_56_H_94_O_24_ | 1195.6080 | -4.2 | -3.5 | +HCOO, -H |
| 19 | Notoginsenoside FP_2_ | 14.89 | C_58_H_98_O_26_ | 1255.6300 | -2.7 | -2.2 | +HCOO, -H |
| 20 | Malnoylfloralginsenosides Rb_1_ | 15.26 | C_57_H_94_O_26_ | 1193.5940 | -2.5 | -2.1 | -H |
| 21 | Notoginsenoside Q | 15.71 | C_63_H_106_O_30_ | 1387.6730 | -2.1 | -1.5 | +HCOO, -H |
| 22 | Vinaginsenoside R_20_ | 15.92 | C_48_H_80_O_20_ | 975.5150 | -2 | -2.1 | -H |
| 23 | Notoginsenoside A | 16.09 | C_54_H_92_O_24_ | 1123.5890 | -1.7 | -1.5 | -H |
| 24 | Ginsenoside F_1_ | 16.41 | C_36_H_62_O_9_ | 683.4376 | 0.0 | 0.0 | +HCOO |
| 25 | Chikusetsusaponin FK_6_ | 16.62 | C_53_H_90_O_22_ | 1123.5900 | -1.0 | -0.9 | +HCOO, -H |
| 26 | Ginsenoside Rs_1_ | 17.3 | C_55_H_92_O_23_ | 1119.5930 | -2.2 | -2.0 | -H |
| 27 | Malnoylfloralginsenosides Rc_1_ | 17.3 | C_56_H_92_O_25_ | 1163.5840 | -1.7 | -1.5 | -H |
| 28 | Malonylginsenoside Rb_2_ | 17.4 | C_56_H_92_O_25_ | 1163.5840 | -1.8 | -1.5 | -H |
| 29 | Pseudoginsenoside F_8_ | 17.68 | C_55_H_92_O_23_ | 1119.5950 | -0.6 | -0.5 | -H |
| 30 | Notoginsenoside K | 18.35 | C_48_H_82_O_18_ | 991.5471 | -1.2 | -1.2 | +HCOO, -H |
| 31 | Malnoylfloralginsenosides Rd_1_ | 18.44 | C_51_H_84_O_21_ | 1031.5410 | -2.3 | -2.2 | -H |
| 32 | Malnoylfloralginsenosides Re_3_ | 18.44 | C_51_H_84_O_21_ | 1031.5420 | -1.6 | -1.5 | -H |
| 33 | Notoginsenoside P | 19.03 | C_52_H_88_O_21_ | 1093.5780 | -2.3 | -2.1 | +HCOO, -H |
| 34 | Floralginsenoside O | 19.42 | C_53_H_90_O_24_ | 1155.5720 | -8.9 | -7.7 | +HCOO |
| 35 | Malnoylfloralginsenosides Rd_6_ | 19.57 | C_54_H_86_O_24_ | 1117.5420 | -1.3 | -1.1 | -H |
| 36 | Quinquefoloside Lb | 19.85 | C_53_H_88_O_22_ | 1075.5680 | -1.9 | -1.8 | -H |
| 37 | Notoginsenoside R_6_ | 19.95 | C_48_H_82_O_19_ | 961.5350 | -2.8 | -2.9 | -H |
| 38 | Notoginsenoside Fe | 20.71 | C_47_H_80_O_17_ | 961.5363 | -1.5 | -1.5 | +HCOO, -H |
| 39 | Ginsenoside Rb_1_ | 20.71 | C_54_H_92_O_23_ | 1107.5940 | -1.7 | -1.5 | -H |
| 40 | Ginsenoside F_2_ | 22.4 | C_42_H_72_O_13_ | 829.4947 | -0.7 | -0.9 | +HCOO |
| 41 | Notoginsenoside ST - _2_ | 22.5 | C_43_H_74_O_15_ | 829.4949 | -0.6 | -0.8 | -H |
| 42 | Ginsenoside Rs_3_ | 22.72 | C_44_H_74_O_14_ | 871.5049 | -1.2 | -1.4 | +HCOO, -H |
| 43 | Ginsenoside Rs_4_ | 23.32 | C_44_H_72_O_14_ | 869.4895 | -0.9 | -1.0 | +HCOO |
| 44 | 24(R) - Pseudoginsenoside F_11_ | 23.72 | C_42_H_72_O_14_ | 799.4845 | -0.4 | -0.5 | -H |
| 45 | Gypenoside XIII | 24.61 | C_41_H_70_O_12_ | 799.4847 | -0.3 | -0.3 | +HCOO, -H |
| 46 | 24(R) - Vinaginsenoside R_1_ | 25 | C_44_H_74_O_15_ | 841.4942 | -1.3 | -1.5 | -H |


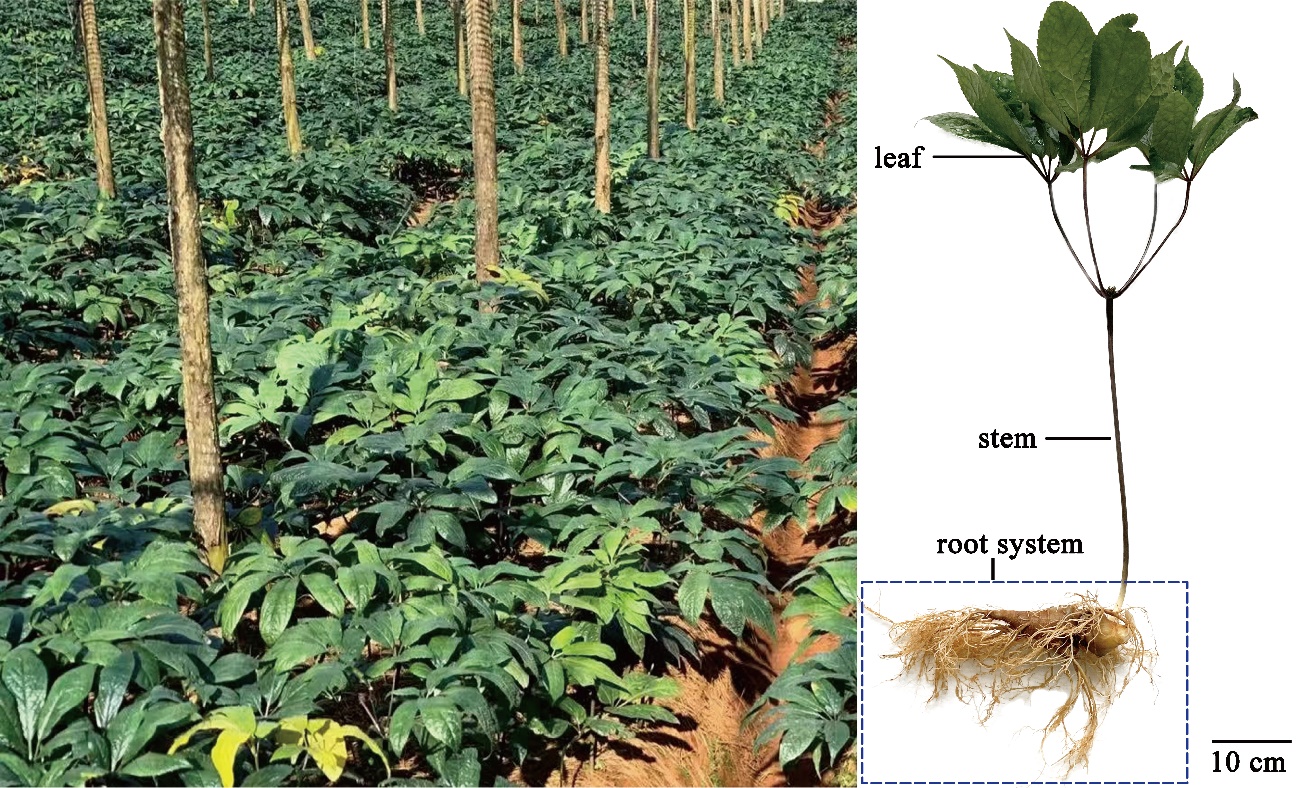


**Figure S1.** Photograph of *Panax notoginseng* plants


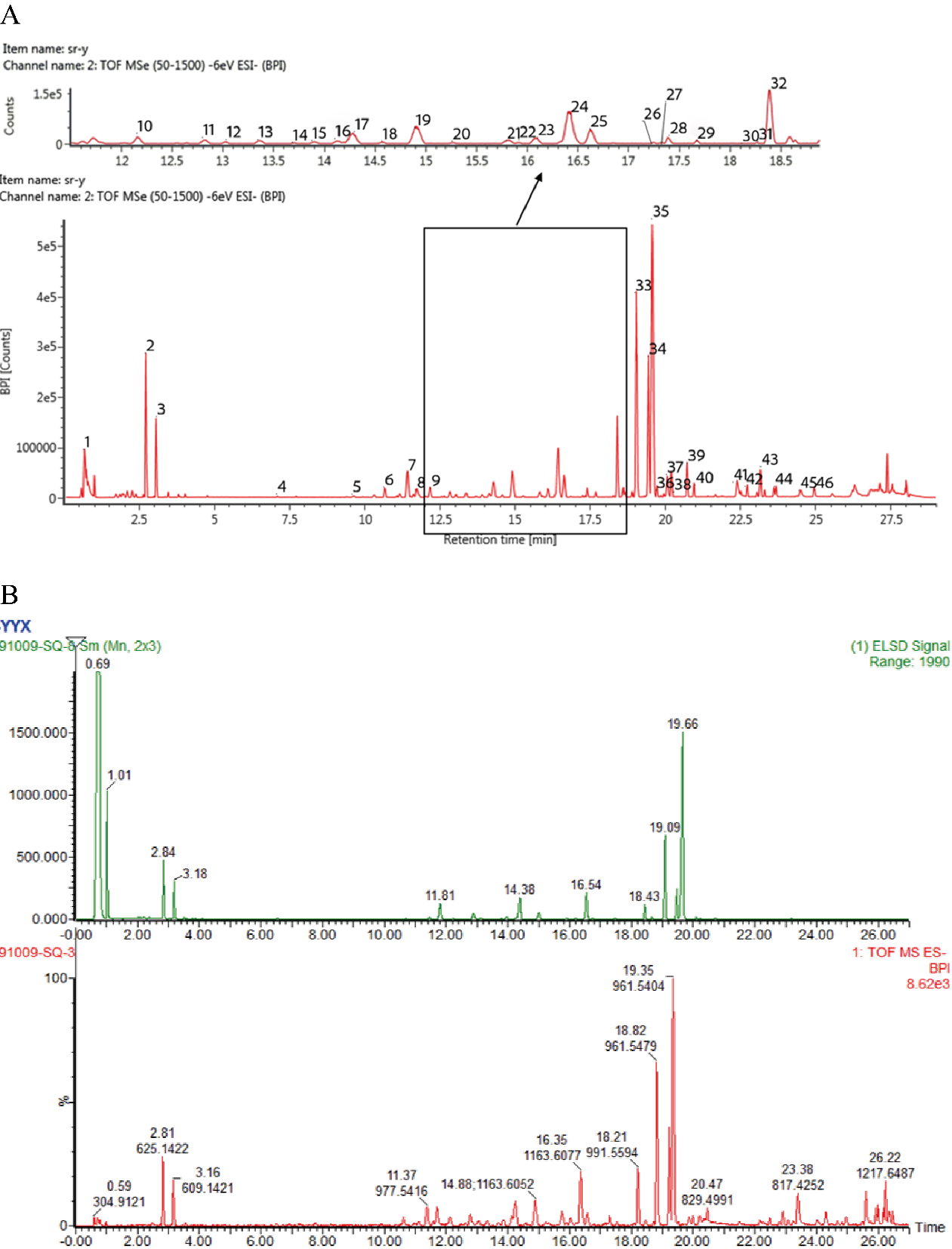


**Figure S2.** Base peak ion chromatogram (BPI) of SLPN analyzed by UPLC - MS (A), principal components of SLPN-instant beverage identified by HPLC-ELSD (B).
